# Supplementary material for: Resolving the full spectrum of human genome variation using Linked-Reads
Source: Genome Res. 2019 Apr;29(4):635–45. doi: 10.1101/gr.234443.118 (PMC6442396; doi:10.1101/gr.234443.118)
Supplement: Supplemental Material [file supp_gr.234443.118_Supplemental_longranger-2.2.zip › longranger-2.2.tar.bz2!/lr2.2-public/lib/stan/cmdstan/stan/doc/api/html/agrad_2fwd_2matrix_2softmax_8hpp_source.html]

Stan: src/stan/agrad/fwd/matrix/softmax.hpp Source File


|  |  |
| --- | --- |
|  | Stan  2.5.0  probability, sampling & optimization |


- Main Page
- Related Pages
- Namespaces
- Classes
- Files

- File List
- File Members

All Classes Namespaces Files Functions Variables Typedefs Enumerations Enumerator Friends Macros Pages

- src
- stan
- agrad
- fwd
- matrix

softmax.hpp

Go to the documentation of this file.

1 #ifndef STAN\_\_AGRAD\_\_FWD\_\_MATRIX\_\_SOFTMAX\_HPP

2 #define STAN\_\_AGRAD\_\_FWD\_\_MATRIX\_\_SOFTMAX\_HPP

3

4 #include <stan/agrad/fwd/fvar.hpp>

5 #include <stan/math/matrix/Eigen.hpp>

6 #include <stan/math/matrix/softmax.hpp>

7

8 namespace stan {

9  namespace agrad {

10

11  template <typename T>

12  inline

13  Eigen::Matrix<fvar<T>,Eigen::Dynamic,1>

14  softmax(const Eigen::Matrix<fvar<T>,Eigen::Dynamic,1>& alpha) {

15  using stan::math::softmax;

16  using Eigen::Matrix;

17  using Eigen::Dynamic;

18

19  Matrix<T,Dynamic,1> alpha\_t(alpha.size());

20  for (int k = 0; k < alpha.size(); ++k)

21  alpha\_t(k) = alpha(k).val\_;

22

23  Matrix<T,Dynamic,1> softmax\_alpha\_t = softmax(alpha\_t);

24

25  Matrix<fvar<T>,Dynamic,1> softmax\_alpha(alpha.size());

26  for (int k = 0; k < alpha.size(); ++k) {

27  softmax\_alpha(k).val\_ = softmax\_alpha\_t(k);

28  softmax\_alpha(k).d\_ = 0;

29  }

30

31  // for each input position

32  for (int m = 0; m < alpha.size(); ++m) {

33  // for each output position

34  T negative\_alpha\_m\_d\_times\_softmax\_alpha\_t\_m

35  = - alpha(m).d\_ \* softmax\_alpha\_t(m);

36  for (int k = 0; k < alpha.size(); ++k) {

37  // chain from input to output

38  if (m == k) {

39  softmax\_alpha(k).d\_

40  += softmax\_alpha\_t(k)

41  \* (alpha(m).d\_

42  + negative\_alpha\_m\_d\_times\_softmax\_alpha\_t\_m);

43  } else {

44  softmax\_alpha(k).d\_

45  += negative\_alpha\_m\_d\_times\_softmax\_alpha\_t\_m

46  \* softmax\_alpha\_t(k);

47  }

48  }

49  }

50

51  return softmax\_alpha;

52  }

53

54

55  }

56 }

57

58 #endif

stan::math::softmax

Eigen::Matrix< T, Eigen::Dynamic, 1 > softmax(const Eigen::Matrix< T, Eigen::Dynamic, 1 > &v)

Return the softmax of the specified vector.

**Definition:** softmax.hpp:46

stan::agrad::fvar

**Definition:** fvar.hpp:13

fvar.hpp

stan::agrad::softmax

Eigen::Matrix< fvar< T >, Eigen::Dynamic, 1 > softmax(const Eigen::Matrix< fvar< T >, Eigen::Dynamic, 1 > &alpha)

**Definition:** softmax.hpp:14

softmax.hpp

Eigen.hpp


---

[ Stan Home Page ]

*© 2011–2014,
Stan Development Team.*
